# CO_2_ Adsorption Behaviors of Biomass-Based Activated Carbons Prepared by a Microwave/Steam Activation Technique for Molecular Sieve

**DOI:** 10.3390/ma16165625

**Published:** 2023-08-15

**Authors:** Jin-Young Lee, Byeong-Hoon Lee, Dong-Chul Chung, Byung-Joo Kim

**Affiliations:** 1 Material Application Research Institute, Jeonju University, Jeonju 55069, Republic of Korea; akdah1tkf@naver.com; 2 Convergence Research Division, Korea Carbon Industry Promotion Agency (KCARBON), Jeonju 54853, Republic of Korea; bhlee@kcarbon.or.kr; 3 Department of Organic Materials & Fiber Engineering, Jeonbuk National University, Jeonju 54896, Republic of Korea; 4 Department of Advanced Materials and Chemical Engineering, Jeonju University, Jeonju 55069, Republic of Korea

**Keywords:** walnut shell, activated carbon, selective adsorption, H_2_/CO_2_, microwave, steam

## Abstract

In this study, the activated carbon was prepared with superior CO_2_ selective adsorption properties using walnut shells, a biomass waste, as a precursor. The activations were conducted at various times using the microwave heating technique in a steam atmosphere. The surface morphology and chemical composition of activated carbon were analyzed using a scanning electron microscope and energy-dispersive X-ray spectroscopy. The textural properties were investigated using the N_2_/77K isothermal method, and the structural characteristics were examined using X-ray diffraction analysis. The CO_2_ and H_2_ adsorption properties of activated carbon were analyzed using a thermogravimetric analyzer and a high-pressure isothermal adsorption apparatus, respectively, under atmospheric and high-pressure conditions. Depending on the activation time, the specific surface area and total pore volume of the activated carbon were 570–690 m^2^/g and 0.26–0.34 cm^3^/g, respectively. The adsorption behaviors of CO_2_ of the activated carbon were different under atmospheric and high-pressure conditions. At atmospheric pressure, a significant dependence on micropores with diameters less than 0.8 nm was observed, whereas, at high pressure, the micropores and mesopores in the range of 1.6–2.4 nm exhibited a significant dependence. However, H_2_ adsorption did not occur at relatively low pressures. Consequently, the prepared activated carbon exhibited superior selective adsorption properties for CO_2_.

## 1. Introduction

The rapid advancement of technology since the industrial revolution in the mid-18th century has caused greater comfort and affluence. However, this progress has led to a significant increase in CO_2_ emissions [1,2]. CO_2_, a prominent greenhouse gas, is a major contributor to global warming, accounting for approximately 75% of greenhouse gas emissions [3]. The increase in global temperatures resulting from global warming has intensified the frequency and severity of natural disasters, including droughts, typhoons, and floods [4,5]. Moreover, they have detrimental effects on the environment and organisms of the earth [6,7]. The international community has realized the severity of global warming caused by CO_2_ and is making efforts to secure CO_2_ removal technologies with superior performance including strengthening CO_2_ emission-related regulations [8]. Furthermore, as the market for fuel cells and automobiles that directly use hydrogen as fuel has expanded, the importance of separating and removing CO_2_ has increased.

Currently, various CO_2_ removal technologies are available, including liquid amine-based absorption processes and those that utilize chemical looping combustion [9,10]. However, these methods suffer from issues such as high energy consumption, high costs, and the potential for secondary pollution [11]. In contrast, activated-carbon-based adsorption technology offers several advantages such as including low cost, superior capture capacity, renewability, and selective adsorption of CO_2_ based on its textural properties [12]. Activated carbon can effectively control atmospheric CO_2_ emissions and possesses selective adsorption properties, making it suitable for application as a carbon molecular sieve (CMS). In particular, high-purity hydrogen, which is currently attracting attention as an energy source, can be separated from a H_2_/CO_2_ mixture [13,14].

Methods of separating specific materials using activated carbon typically include pressure swing adsorption (PSA) and temperature swing adsorption (TSA). Among them, the PSA method was devised by Skarstrom in the 1960s and applied to separation processes such as air separation and hydrogen purification due to low energy requirements compared to other process methods [15,16,17]. The performance of the PSA process depends on the selective adsorption characteristics of the adsorbent, and zeolite has been mainly studied as an adsorbent widely used in the PSA process [18]. However, zeolite has limited separation performance due to its relatively low selective adsorption performance [18]. Therefore, research on activated carbon and metal organic frameworks (MOFs) has been conducted to find adsorbents more suitable for the PSA process (advantages and disadvantages of each adsorption material are shown in Table S1) [19,20,21]. MOF has a high CO_2_ adsorption capacity and a high specific surface area, but it is limited in its application to PSA based on expensive synthesis processes [19,22]. Activated carbon, another adsorbent, can be a good solution for the PSA process based on advantages such as a wide range of precursors, relatively low costs, and low energy consumption [19,20,21]. The adsorption of CO_2_ by activated carbon is mainly due to physical adsorption based on Van der Waal’s interaction, and the CO_2_ adsorption performance increases as the pressure increases [19]. However, the post-combustion gas is emitted at a temperature in the range of 40 to 160 °C and an overall pressure of about 1 atm, where CO_2_ has a relatively low partial pressure of 0.05–0.15 atm [23]. Therefore, activated carbon with excellent selective adsorption performance of CO_2_ at relatively low pressure is required. 

Lee et al. [24] successfully prepared high-quality microporous activated carbon using a chemical activation method involving KOH. This method produces activated carbon with outstanding CO_2_ adsorption performance. However, chemical activation has limitations that hinder its practical applications in industrial settings. These disadvantages include the need for an additional washing process, increased processing costs due to the generation of chemical residues, and the potential for equipment corrosion [25]. In contrast, physical activation offers an eco-friendly alternative because it does not generate chemical residues. In addition, physical activation is well suited for large-scale production of activated carbon in industrial fields because of its simplicity and the lack of equipment damage [26].

The commonly employed electric-resistance-heating furnace method for activation relies on conduction, radiation, and convective energy transfer. However, it has the disadvantage of high energy consumption because the processing time is extended owing to the relatively low heat-transfer rate of the material. Alternatively, researchers have employed the microwave heating method [27,28]. This method involves ion conduction and dipole rotation, where microwaves are directed toward the material, transforming them into heat within the material itself. This enables rapid heating and reduces the processing time and energy consumption [29].

In this study, activated carbon was prepared from walnut shells, a biomass waste, through efficient energy consumption and eco-friendly activation processes. Various microwave heating activation times were employed to control the textural properties of walnut shell-based activated carbon. The effect of changes in the structure of activated carbon over the activation time on the textural properties of activated carbon was evaluated. Further, changes in the textural properties of activated carbon were examined through changes in the pore volume and diameter. The CO_2_ and H_2_ adsorption capacities of the prepared activated carbon according to pressure were also analyzed to determine the applicability of H_2_/CO_2_ separation in the PSA process.

## 2. Experimental Methods

### 2.1. Preparation of Activated Carbon

In this study, walnut shells were obtained from commercial walnuts as activated carbon precursors. Walnut shells were dried at 105 °C for more than 24 h. To carbonize the walnut shell, 10 g of the walnut shell was filled in an alumina boat, placed in a cylindrical quartz tube (diameter 90 mm × length 1000 mm), heated to 800 °C at a heating rate of 10 °C/min in an N_2_ (200 mL/min) atmosphere, and maintained for 1 h. After heating, this was followed by natural cooling to 30 °C. The yield of carbonized walnut shells was observed to be 29.0%

To proceed with the activation, the walnut shell char was pulverized and sieved to less than 2 mm (Figure S1 shows the particle size of walnut shell-based activated carbon). Then, 3 g of the sieved walnut shell char was filled into a home-made quartz reactor (diameter 70 mm × length 100 mm). The quartz reactor was placed in a modified microwave oven (MW22CD9D, LG Electronics, Seoul, Korea). Before activation, the quartz reactor was degassed with N_2_ (200 mL/min) gas. Activation was performed for 10–25 min at 1 kW with the flow rate set to H_2_O (0.1 mL/min). After activation, the gas flow was changed from H_2_O to N_2_ (200 mL/min), and the sample was cooled to 30 °C. The activation reaction between the carbon crystallites and H_2_O is an endothermic reaction in stoichiometric form, as shown in Equation (1).

C + H_2_O → CO + H_2_    ΔH = +117 kJ/mol (1)

A schematic of the microwave steam activation technique used in this study is indicated in Figure S3. Each sample name was a walnut shell (WS)-microwave (M) activation time (walnut shell-based activated carbon activated by microwave for 20 min: WS-M20).

### 2.2. Characterization

Scanning electron microscope (SEM; S-4800, HITACHI, Tokyo, Japan) and energy-dispersive X-ray spectroscopy (EDX; EMAX, HORIBA, Kyoto, Japan) were used for the surface morphology and elemental analysis of the walnut shell-based activated carbon (WS-AC). In the SEM-EDX analysis, WS-AC was coated with platinum to reduce charging and to obtain clear images under operating voltage and pressure conditions of less than 10 kV and 1 × 10^−5^ Torr, respectively.

The crystal structure of WS-AC was investigated using X-ray diffraction (XRD; Empyrean, Malvern Panalytical, Malvern, UK). XRD patterns were recorded in the 2θ range of 10–60° at a rate of 2°/min using Cu Kα (1.542 Å) radiation. The crystallite size and interplanar spacing of WS-AC were calculated using the Scherrer’s equation and Bragg’s equation, respectively [30].

The textural properties of WS-AC were analyzed using N_2_/77K adsorption–desorption isotherms obtained by BELSORP-Max II (Microtrac BEL, Osaka, Japan) and pretreated at a pressure of less than 1 × 10^−3^ Torr. The specific surface area and micropore volume of the activated carbon were calculated using the Brunauer–Emmett–Teller’s equation [31] and the t-plot method [32], respectively. The nonlocalized density functional theory (NLDFT) method was used to determine the pore-size distribution of WS-AC [33,34].

### 2.3. CO_2_ and H_2_ Adsorption Behaviors

Thermogravimetric analysis (TGA; TGA/DSC 3+, Mettler-Toledo, Columbus, OH, USA) was used to evaluate the CO_2_ adsorption performance of WS-AC at atmospheric pressure. Briefly, in the analyzer, 10 mg of WS-AC was placed in a 70 mL alumina pan and degassed at 110 °C in an N_2_ (50 mL/min) atmosphere until no change in weight occurred. Then, after cooling to 25 °C the gas flow inside the analyzer was changed from N_2_ to CO_2_ (50 mL/min), and the weight change in activated carbon was evaluated corresponding to the CO_2_ adsorption behavior.

The high-pressure gas adsorption system, BELSORP-HP (Microtrac BEL, Osaka, Japan) was used to evaluate the CO_2_ and H_2_ adsorption performances of WS-AC at high pressure. Before the analysis, 0.5 g of all WS-ACs were treated at a temperature of 300 °C and a pressure of less than 1 × 10^−3^ Torr. The measurement pressure range was 0–30 bar, and the temperature was maintained at 25 °C (in the PSA process, adsorption was performed five times to confirm the stability of CO_2_ adsorption circulation of activated carbon and is shown in Figure S2).

## 3. Results and Discussion

### 3.1. Surface Morphology and Chemical Composition

**Figure 1:**
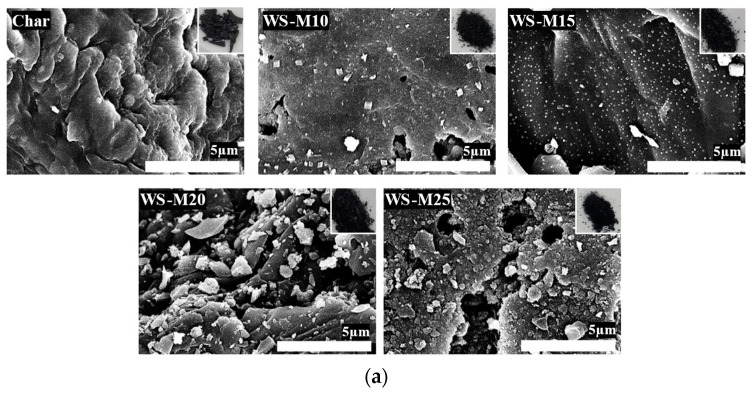

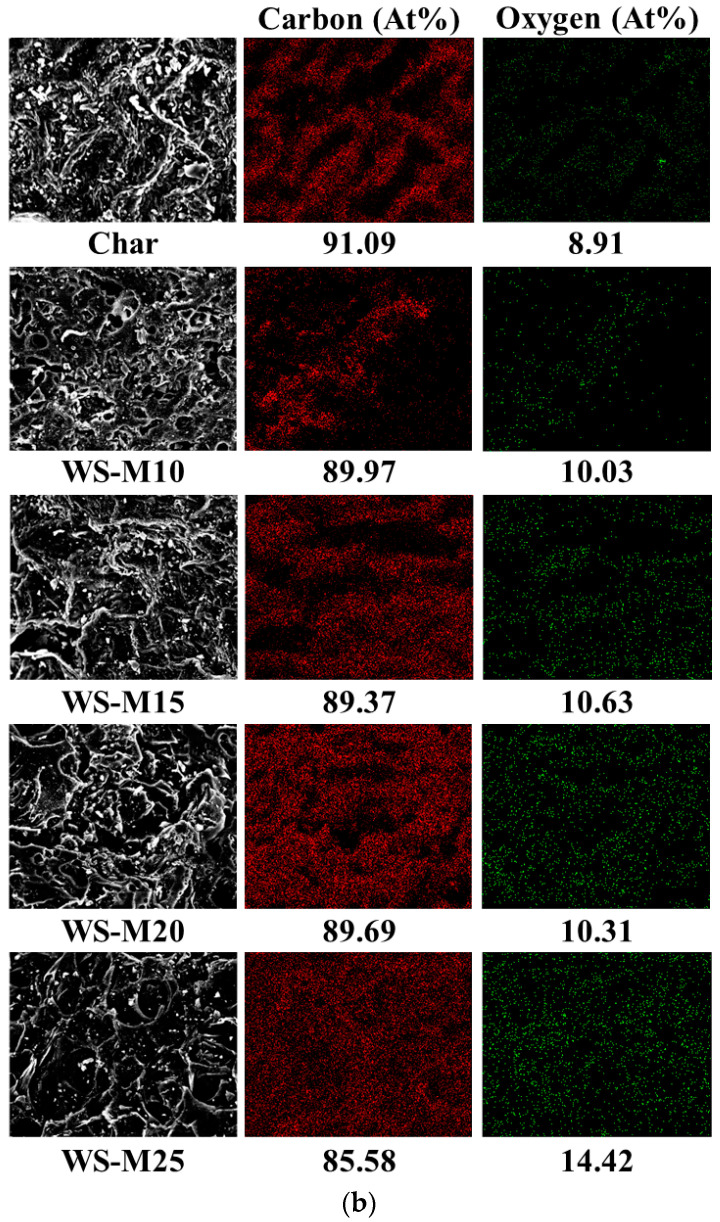
Scanning electron microscope image (**a**) and energy-dispersive X-ray spectroscopy mapping image (**b**) of walnut shell-based activated carbons as a function of activation time.

The optical and SEM images of WS-AC are exhibited in Figure 1a. In the optical image, the color of the walnut shell (WS) is black, which is attributed to the conversion of WS into a material with an abundant carbon content through decomposition, polycondensation, and cyclization during the carbonization process. The SEM image exhibits that surface pores were formed on the smooth surface of the char, and the roughness increased as the activation progressed. This was attributed to the decomposition of the carbon structure generated by steam at high temperatures during the microwave heating process.

Figure 1b shows the EDX mapping images of WS-AC. All WS-ACs have a high carbon content of 85.58–91.09%, which is also attributed to the conversion of walnut shells to materials with a high carbon content. Further, as the activation time increased, the carbon content of WS-AC decreased, and the oxygen content increased. Nabais et al. [35] confirmed that the carbon content decreased, and the oxygen content increased as the activation process intensified in activated carbon, which is ascertained to the formation of functional groups. Therefore, the decrease in the carbon content and increase in the oxygen content of WS-AC are an effect of activation, resulting in the decomposition of the carbon structure and the consequent formation of oxygen functional groups.

### 3.2. Textural Properties and Structure 

**Figure 2:**
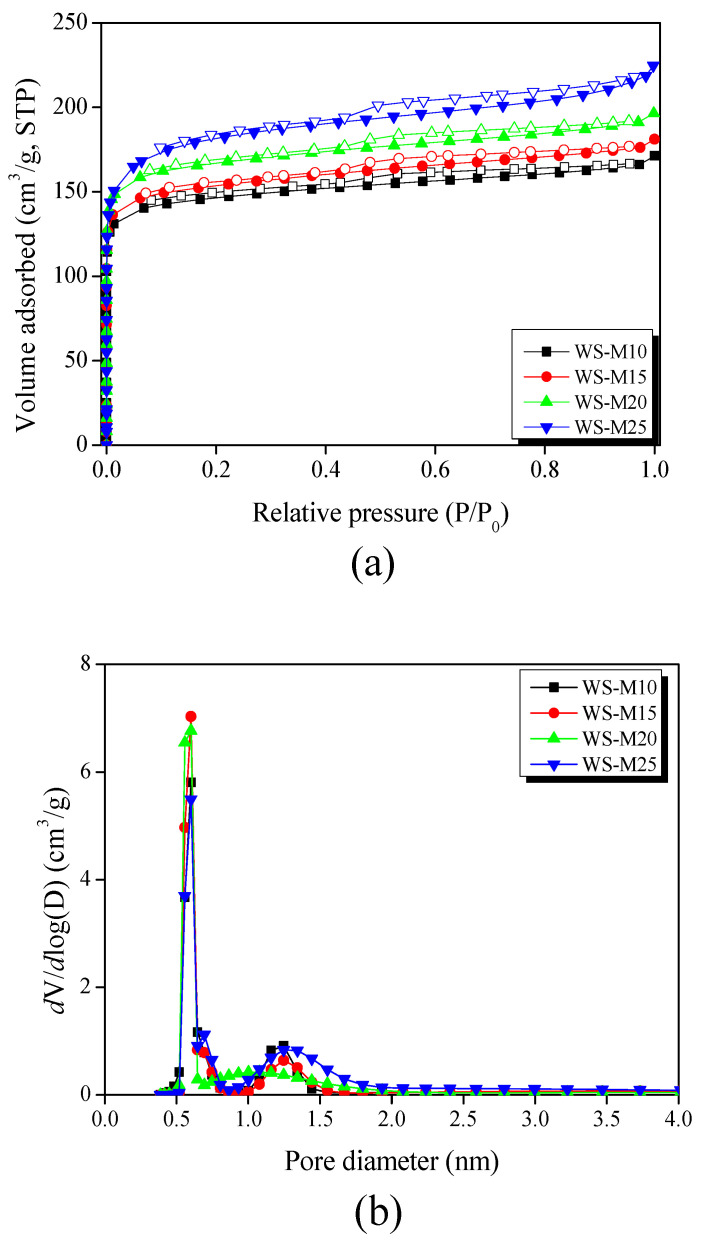

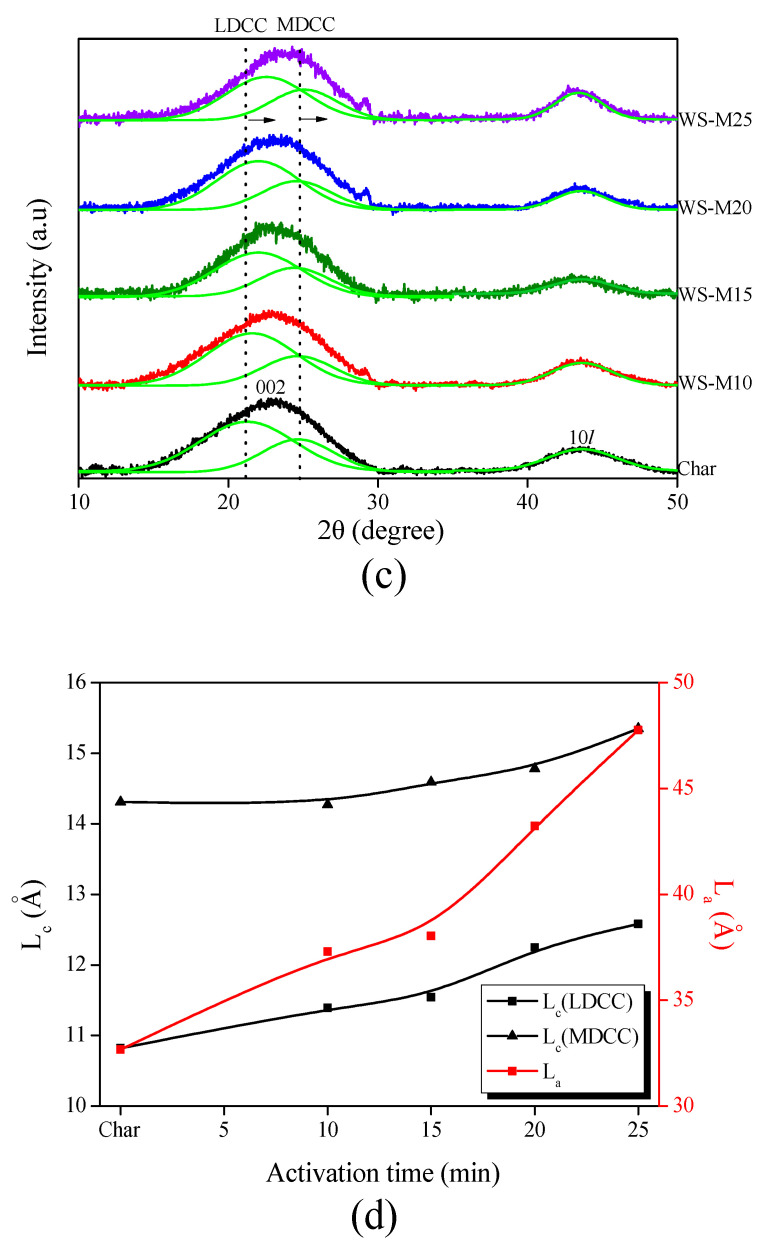
(**a**) N_2_/77K adsorption–desorption isotherm, (**b**) pore size distribution by NLDFT, (**c**) deconvoluted X-ray diffraction pattern, and (**d**) crystallites size of walnut shell-based activated carbons as a function of activation time.

The N_2_/77K adsorption–desorption isotherm is a powerful tool for analyzing the pore structure of activated carbon. Figure 2a exhibited the N_2_/77K adsorption–desorption isotherms of WS-AC as a function of activation time. All WS-ACs were categorized as Type I by the IUPAC classification [36], which primarily consists of micropores. As the activation time increased, N_2_ adsorption gradually increased at a relative pressure (P/P_0_) of less than 0.1, and the hysteresis also increased. This could be owing to the development of micropores due to the oxidation of amorphous and small crystallites. Further, as the activation time increased, the wedge-shaped micropores became pot-type mesopores owing to the oxidation of the edges of crystallite (Table S2 lists the textural properties parameter of walnut shell-based activated carbon).

Figure 2b shows the pore size distribution of WS-AC investigated using the NLDFT method. WS-AC comprised pores with diameters of 1 nm or less and pores with diameters of approximately 1–2 nm. Pores less than 1 nm in diameter gradually developed until 20 min of activation time but decreased at 25 min. Meanwhile, the diameters of the 1–2 nm pores gradually expanded and developed as the activation time increased. As the amorphous is decomposed by activation, narrow micropores developed, and as the activation proceeds, these micropores become wide owing to expansion in diameter and increase in volume due to the small crystallite oxidation and greater decomposition of the amorphous.

XRD is a useful analytical tool for examining changes in the crystal structure of activated carbon as a function of activation time. In Figure S4, the XRD patterns of WS-AC exhibit the 002 peak (approximately 23–24°), which is different from the 002 peak (26.56°) of graphite. Furthermore, a broad 10l peak (approximately 43°), which cannot be separated, was observed and was attributed to the effect of the disordered graphite-like structure and irregularly stacked carbon layer.

Figure 2c exhibits the XRD deconvolution curves of WS-AC according to the activation conditions. The 002 peaks were deconvoluted using Gaussian fitting and separated into less-developed crystalline carbon (LDCC) and more-developed crystalline carbon (MDCC). Meanwhile, the correlation coefficient of the deconvolved peak is more than 0.99 (the structural parameters are listed in Table S3). In WS-AC, LDCC and MDCC, constituting the 002 peaks, shifted in a direction closer to that of crystallinity as the activation time increased, including char, and the change in LDCC was observed more clearly than that of MDCC. Physical activation is preceded by the development of micropores due to the decomposition of amorphous and small crystallites. As the activation continues, the edges of large crystallites constituting the carbon domain are oxidized to develop into mesopores. Therefore, the change in the XRD pattern is due to the amorphous phase preferentially decomposed by physical activation and it is considered that a tendency to improve crystallinity has been observed.

Figure 2d exhibited the change in L_c_ (LDCC and MDCC) and L_a_ of WS-AC according to the activation time calculated from the XRD deconvoluted curve. A tendency to in-crease both L_a_ and L_c_ was observed with increasing activation time. In addition, the change in the lateral size (L_a_) was greater than the change in the stacking height (L_c_). This is because the layer of the (002) plane in the crystal structure is composed of strongly hybridized sp^2^ bonds, and the vertical π-bond on the (002) plane results in weak interlayer bonds [37]. Furthermore, a relatively larger change in the L_c_ of LDCC than that of MDCC was observed, which is considered to be an effect of the preferential decomposition of LDCC. Lee et al. [38] reported that upon physical activation, the amorphous region is preferentially decomposed, the crystallites size increases, and micropores develop. Accordingly, it is considered that the change in the crystallite size of WS-AC is not due to the growth of carbon crystallites, but rather the increase in the relative crystallite size due to the decomposition of amorphous regions and the oxidation of small crystallites [39,40]. Therefore, it is expected that the micropores of WS-AC develop due to the decomposition of the amorphous region of the carbon structure, and as the activation progresses, the small crystallites are oxidized, and the pore diameter expands (schematic diagram of the change in the crystal structure of carbon by steam activation is shown in Figure S5).

### 3.3. Adsorption Behaviors and Correlation

**Figure 3:**
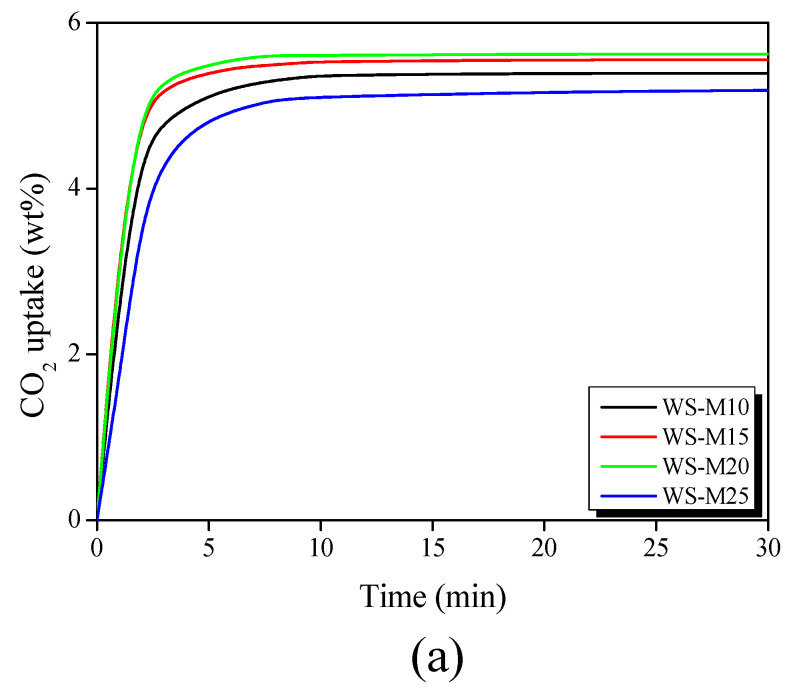

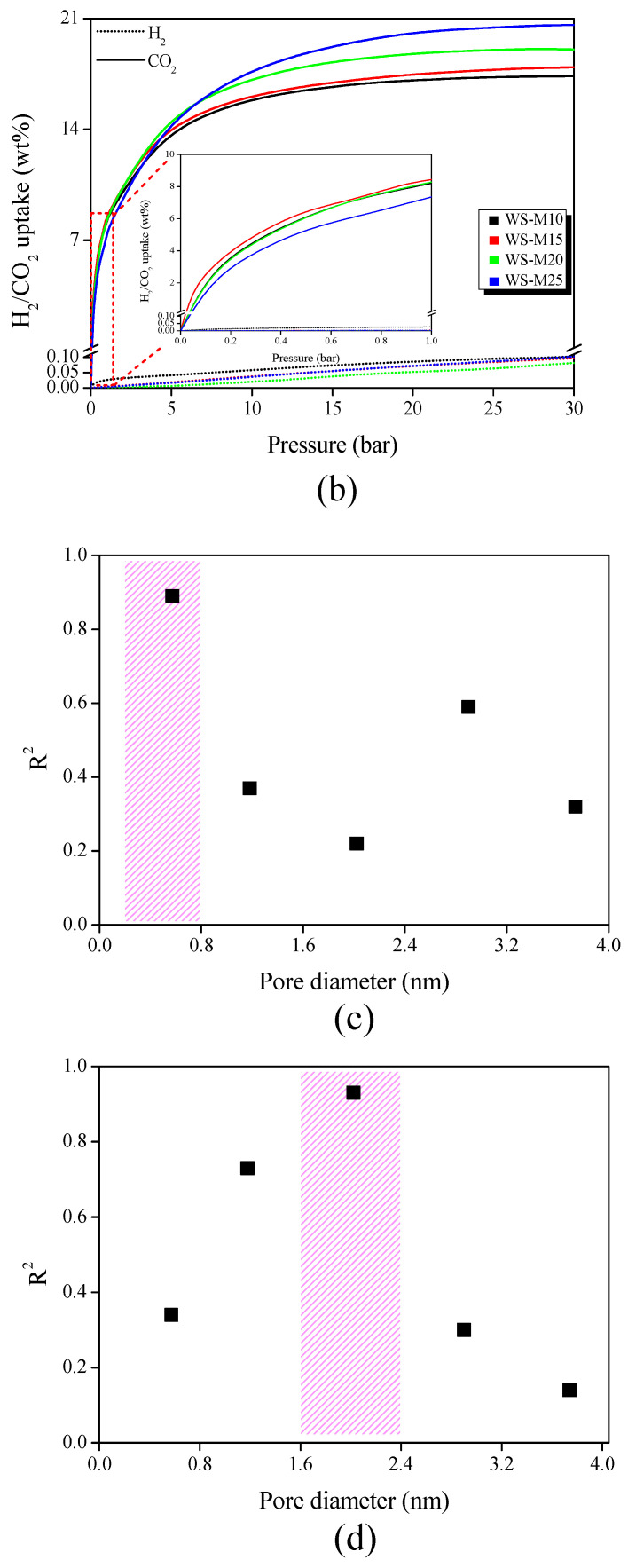
(**a**) CO_2_ storage capacity of walnut shell-based activated carbons at 1 bar/298K, (**b**) CO_2_ and H_2_ storage capacity of walnut shell-based activated carbons up to 30 bar, (**c**) correlation coefficient between pore diameters and CO_2_ capacity at 1 bar, and (**d**) correlation coefficient between pore diameters and CO_2_ capacity at 30 bar.

Figure 3a shows the CO_2_ adsorption curve of WS-AC at atmospheric pressure, obtained using a TGA. The CO_2_ adsorption capacities are recognized in the order of WS-M20 > WS-M15 > WS-M10 > WS-M25. In addition, the adsorption rate, according to the slope of the CO_2_ adsorption curve, was in the order of WS-M20 > WS-M15 > WS-M10 > WS-M25, which was the same as the adsorption capacity. In all WS-ACs, the adsorption equilibrium was observed for CO_2_ at approximately 10 min. Lee et al. [24] and Jagiello et al. [25] reported that the effective pore diameter of activated carbon for the adsorption of CO_2_ at atmospheric pressure was less than 0.8 nm. The pore diameter distribution of WS-AC is shown in Figure 2b, and WS-20 has the most pores with a diameter of less than 1 nm. This is based on the micropore filling theory, in which the CO_2_ adsorption performance of WS-AC is preferentially adsorbed in micropores with diameters less than 1 nm, and the diameter of pores effective for CO_2_ adsorption might increase as the pressure increases [39]. Therefore, activated carbon requires the development of narrow micropores with a diameter of less than 1 nm to achieve superior CO_2_ adsorption performance at atmospheric pressure.

Figure 3b exhibits the adsorption curves of CO_2_ and H_2_ of WS-AC measured at 0–30 bar conditions. The aforementioned CO_2_ adsorption behavior was similar to the CO_2_ adsorption behavior under atmospheric pressure conditions (Figure 3a) and 1 bar however, the CO_2_ adsorption capacity under a pressure of further or more was different from the CO_2_ adsorption under atmospheric pressure. In general, under atmospheric pressure, the filling of the narrow micropore area precedes and saturates, and as the pressure increases, wide micropores and narrow mesopores are filled. Casco et al. [41] reported that under 1 MPa conditions, activated carbon is almost saturated in narrow micropores and requires a higher pressure to cause adsorption in wider pore diameters. Accordingly, WS-M25, which has numerous wide micropores and narrow mesopores, exhibits superior CO_2_ capacity compared to the other activated carbon samples under high-pressure conditions. In the case of H_2_ adsorption, an extremely small amount of H_2_ was adsorbed on all WS-ACs. Lee et al. [37] reported that the hydrogen storage capacity of activated carbon was affected by the micropore volume, and also inferred that the micropores in the range of 0.63–0.78 nm, controlled hydrogen adsorption under 25 °C and 10 MPa conditions. These results substantiate that a higher pressure is required or that the pore diameter of the activated carbon needs to be further reduced for WS-AC to adsorb hydrogen. Therefore, WS-AC is expected to be useful for separating high-purity H_2_ by selective adsorption of CO_2_ from the H_2_/CO_2_ mixture when applied as a CMS in the PSA process.

Figure 3c shows the correlation between the CO_2_ adsorption capacity and the pore diameter of WS-AC under atmospheric pressure. The NLDFT data in Figure 2b were used as the pore diameter data. CO_2_ adsorption at atmospheric pressure exhibited a high correlation (R^2^ = 0.89) with pores having a diameter of 0.8 nm or less. CO_2_ adsorption is mainly owing to Van der Waal’s interactions (physical adsorption) and is most suitable when the pore diameter is approximately two to three times that of the adsorbate [25]. Therefore, at atmospheric pressure, WS-AC can remove CO_2_ molecules through strong interactions with pores with a diameter of 0.8 nm or less. 

Figure 3d shows the correlation between the CO_2_ capacity and diameter of WS-AC at 30 bar. CO_2_ adsorption under high pressure has a high correlation (R^2^ = 0.93) with wide micropores and narrow mesopores with diameters of 1.6–2.4 nm, and pore regions with other diameters show a low correlation. Carsco et al. [41] reported that 4.5 MPa was not sufficient to fill mesopores larger than 3.0 nm and were not suitable for capillary condensation to occur. Therefore, pores with diameters of 1.6–2.4 nm generated capillary condensation in WS-AC under 30 bar conditions, and it is effective in the adsorption of CO_2_.

### 3.4. Fitting of CO_2_ Adsorption Isotherms

**Figure 4:**
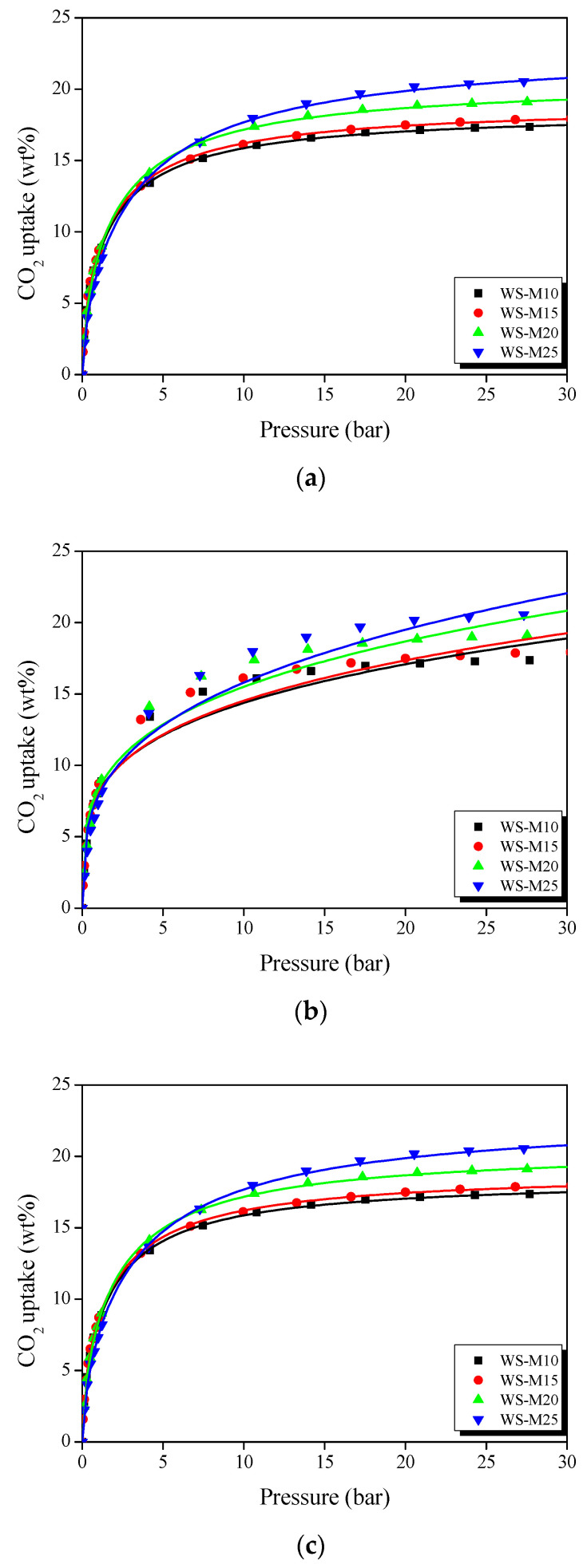
Comparison of the fitting results by the (**a**) Langmuir, (**b**) Freundlich, and (**c**) Langmuir–Freundlich equations for the adsorption of CO_2_ on walnut shell-based activated carbon.

The Langmuir [42], Freundlich [43], and Langmuir–Freundlich [44] models were adopted to explain the CO_2_ adsorption behavior according to the pressure of activated carbon, calculated by Equations (2)–(4), shown in Figure 4a–c, respectively (Table S4 showed parameters according to Isotherm model star).

(2) $$q=\frac{q_{m}K_{L}P}{1+K_{L}P}$$

(3) $$q=K_{F}P^{\frac{1}{n}}$$

(4) $$q=\frac{q_{m}K_{T}P^{\frac{1}{n}}}{1+(K_{L}P)P^{\frac{1}{n}}}$$

K_L_ is the Langmuir isotherm constant (bar^−1^), q_m_ is the maximum adsorption capacity, P is the relative pressure of the adsorbate (bar), K_F_ is the Freundlich isotherm constant (bar^−1^), and n is the Freundlich exponent.

The Langmuir model is based on monolayer adsorption on a homogeneous surface, and the Freundlich model is based on multilayer adsorption on a heterogeneous surface. The Langmuir–Freundlich model is a model that combines the previous two models. The Langmuir model exhibited a high fit (R^2^: 0.99), but the Freundlich model indicated a relatively low fit (R^2^: 0.96). It is inferred that the CO_2_ adsorption behavior of WS-AC with narrow micropores is mainly adsorbed by the monolayer by filling the micropores. Also, as the pressure increases, the adsorption of CO_2_ is considered to be caused by additional multilayer adsorption. Accordingly, the highest fit (R^2^: 0.9999) was observed in the Langmuir–Freundlich model, which is a combination of the two models. Therefore, it can be expected that the CO_2_ behavior of activated carbon mainly occurs in the relatively low pressure part by monolayer adsorption by filling micropores, and multilayer adsorption occurs as the pressure increases.

### 3.5. Comparison of CO_2_ Capture Capacity of Biomass Activated Carbon

To assess the applicability of the activated carbon used in this study, a comparison was made with biomass-based activated carbon produced in other research, and the results were presented in Table 1. In the case of physical activation, activated carbon produced by conventional heating methods showed similar or up to 1.6 times enhanced CO_2_ adsorption performance compared to microwave treatment. However, the activation time for these conventional methods ranged from 60 to 240 min, which is 3 to 12 times longer than the method used in this study. This leads to significant energy consumption and a subsequent decrease in economy. For chemical activation, the CO_2_ performance was enhanced up to two times compared to physical activation. However, despite relatively shorter activation times compared to microwave methods, chemical activation still required 3 to 4.5 times longer activation periods. Additionally, chemical activation has drawbacks such as wastewater generation, equipment corrosion, and increased process costs due to additional washing steps. Therefore, eco-friendly activated carbon in this study can reduce process costs due to fast activation time, and problems arising from chemical activation do not occur, so it is considered to be a superior adsorbent for CO_2_ capture in industrial processes.

## 4. Conclusions

In this study, activated carbon was prepared from walnut shells using a microwave heating technique with low energy consumption and steam, an eco-friendly activator, and the adsorption behavior of CO_2_ and H_2_ according to pressure was investigated to determine the activated carbon suitable for the PSA process. The microwave/steam activation technique followed a typical physical activation mechanism, and through analysis of the crystal structure and textural properties of activated carbon, it was confirmed that micropores were developed due to preferential decomposition of amorphous. WS-AC showed excellent selective CO_2_ adsorption performance and hydrogen separation in all pressure ranges, especially in the relatively low pressure range. Narrow micropores of 0.8 nm or less were effective in CO_2_ adsorption at atmospheric pressure, and effective pore diameter for CO_2_ adsorption at high pressure (30 bar) was 1.6 to 2.4 nm. Therefore, WS-AC manufactured as an eco-friendly activation means from walnut shells, which are biomass waste, can be a good alternative as an adsorbent for the PSA process.

## Figures and Tables

**Table 1:** Comparison of activation method and CO_2_ adsorption capacity of biomass-based activated carbons.

Sample	CO_2_ Adsorption Amount (mmol/g) at 298 K and 1 Bar	Heating Method	Activation Time (min)	Ref.
Walnut shell	1.9	Microwave (physical)	20	Our work
Coconut shell	1.8	Conventional heating (physical)	60	[45]
Almond shell	2.7	Conventional heating (physical)	240	[46]
Olive stone	2.9	Conventional heating (physical)	240	[46]
Coffee residue	2.4	Conventional heating (physical)	60	[47]
Almond shell	2.1	Conventional heating (physical)	83	[48]
Olive stone	2.0	Conventional heating (physical)	110	[48]
Amazonian waste	3.7	Conventional heating (Chemical)	60	[49]
Cotton stalk	2.9	Conventional heating (Chemical)	90	[50]
Coconut shell	2.6	Conventional heating (Chemical)	60	[51]
DARCO FGD(Norit)	0.4 (291 K, l bar)	-	-	[52]

## Data Availability

Not applicable.

## Supplementary Materials

The following supporting information can be downloaded at: https://www.mdpi.com/article/10.3390/ma16165625/s1, Table S1: Advantages and disadvantages of CO_2_ adsorption by adsorbent; Figure S1: Particle size distribution of walnut shell-based activated carbon; Figure S2: CO_2_ adsorption curve of activated carbon according to five times; Figure S3: Schematic diagram of microwave steam activation system; (a) nitrogen storage, (b) H_2_O-micro feeder, (c) regulator, (d) heating zone temperature controller, (e) heating zone, (f) valve, (g) flow meter, (h) quartz reactor, (i) walnut shell char, (j) microwave oven, (k) trap, and (l) vent. Figure S4: Original X-ray diffraction of walnut shell-based activated carbon as a function of activation time; Figure S5: Schematic diagram of carbon structure changes by steam activation; Table S2: Textural properties of walnut shell-based activated carbons as a function of activation time; Table S3: Structural parameters of walnut shell-based activated carbon as a function of activation time; Table S4: The correlation parameters by adsorption isotherms model.
