# Supplementary material for: CO2 Adsorption Behaviors of Biomass-Based Activated Carbons Prepared by a Microwave/Steam Activation Technique for Molecular Sieve
Source: Materials (Basel). 2023 Aug 15;16(16):5625. doi: 10.3390/ma16165625 (PMC10456295; doi:10.3390/ma16165625)
Supplement: Supplementary file 1 [file materials-16-05625-s001.zip › materials-2554327-supplementary.pdf]

CO_2 Adsorption Behaviors of Biomass-Based Activated Carbons Prepared by a Microwave/Steam Activation Technique for Molecular Sieve

Jin-Young Lee [1], Byeong-Hoon Lee [2], Dong-Chul Chung [3,*] and Byung-Joo Kim [1,4,*]

[1] Material Application Research Institute, Jeonju University, Jeonju 55069, Republic of Korea; akdah1tkf@naver.com
[2] Convergence Research Division, Korea Carbon Industry Promotion Agency (KCARBON), Jeonju 54853, Republic of Korea; bhlee@kcarbon.or.kr
[3] Department of Organic Materials & Fiber Engineering, Jeonbuk National University, Jeonju 54896, Republic of Korea
[4] Department of Advanced Materials and Chemical Engineering, Jeonju University, Jeonju 55069, Republic of Korea
* Correspondence: sonagiii0523@gmail.com (D.-C.C.); kimbyungjoo@jj.ac.kr (B.-J.K.)

Table S1. Advantages and disadvantages of CO_2 adsorption by adsorbent

Adsorbent material	Advantages	Disadvantages
Activated carbon	low cost, high specific surface area, easy low energy consumption, wide range of precursors	friability, price rise according to precursor.
Zeolite	high CO_2 adsorption capacity, high surface area and porosity	low selectivity, expensive synthesis
Metal organic frameworks	high CO_2 adsorption capacity	complex and expensive synthesis, low tolerance to moisture/impurities

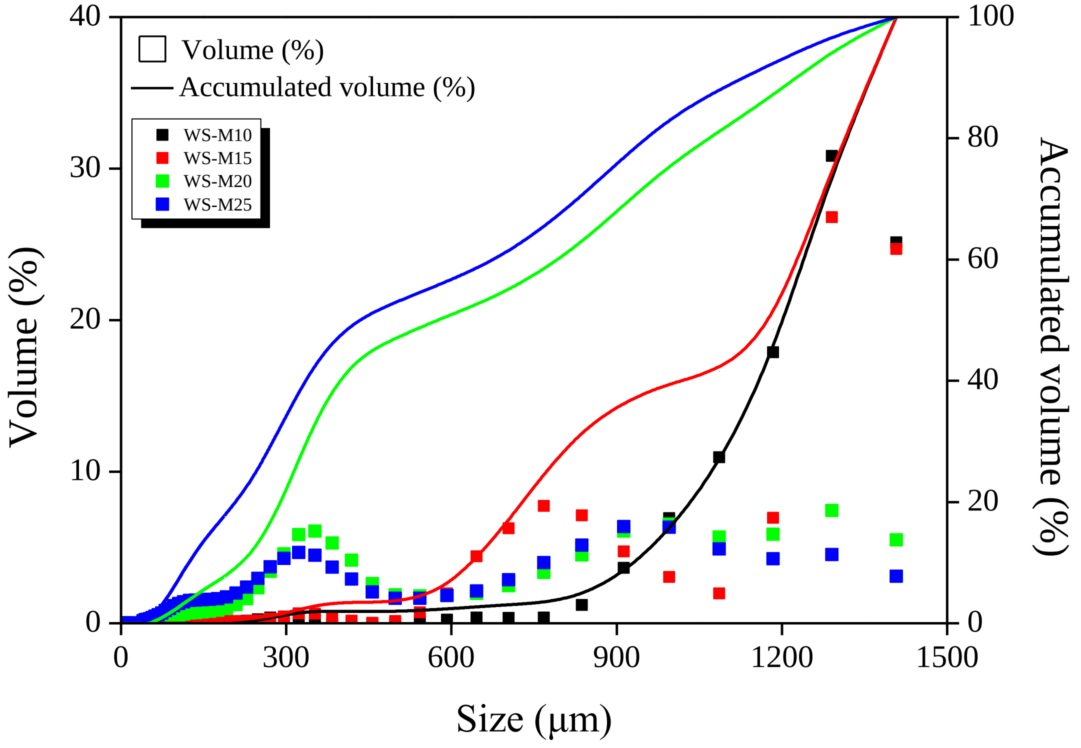

Figure S1. Particle size distribution of walnut shell-based activated carbon.

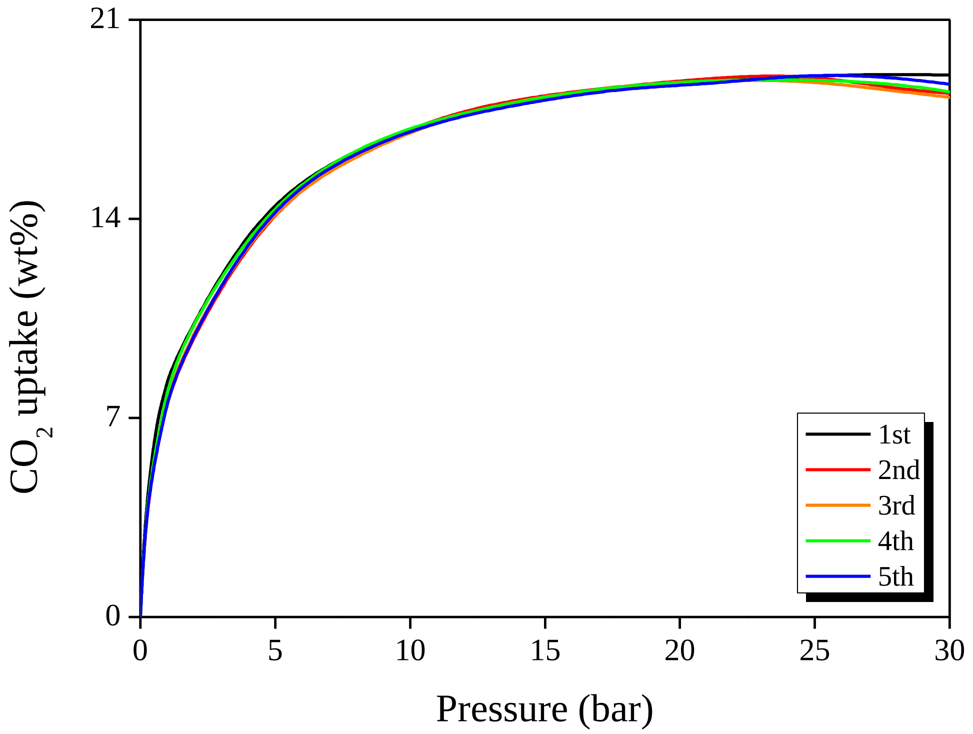

Figure S2. CO_2 adsorption curve of activated carbon according to five times.

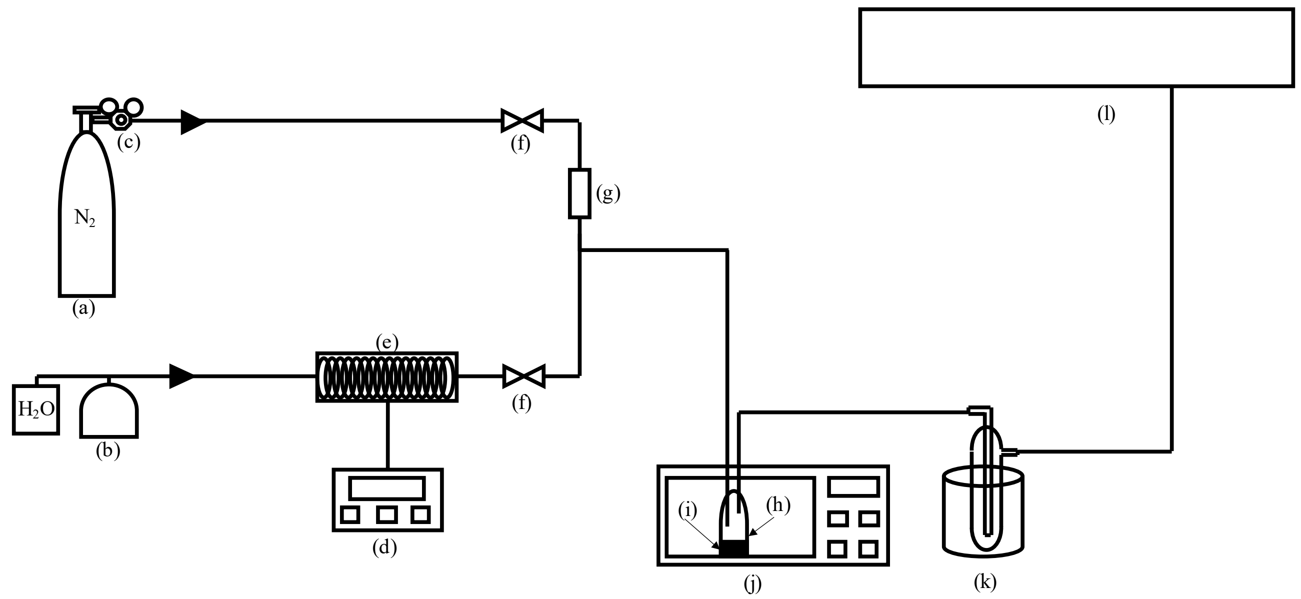

Figure S3. Schematic diagram of microwave steam activation system; (a) nitrogen storage, (b) H_2O-micro feeder, (c) regulator, (d) heating zone temperature controller, (e) heating zone, (f) valve, (g) flow meter, (h) quartz reactor, (i) walnut shell char, (j) microwave oven, (k) trap, and (l) vent.

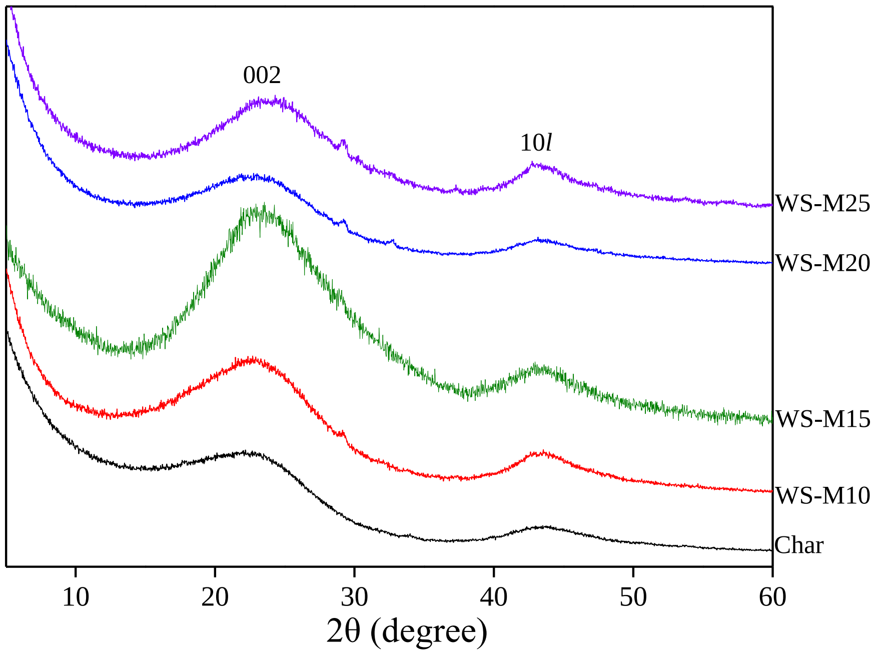

Figure S4. Original X-ray diffraction of walnut shell-based activated carbon as a function of activation time**.**

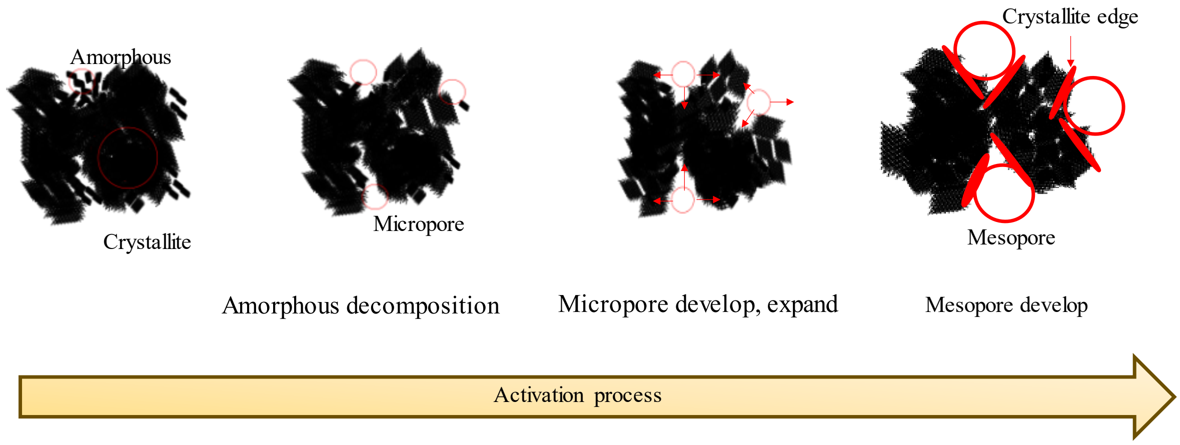

Figure S5. Schematic diagram of carbon structure changes by steam activation.

Table S2. Textural properties of walnut shell-based activated carbons as a function of activation time

Sample	S_{BET} (m^2/g)	V_{Total} (cm^3/g)	V_{Micro} (cm^3/g)	V_{Meso} (cm^3/g)	Micropore ratio (%)	Yield (%)
WS-M10	570	0.26	0.22	0.04	84.6	19.3
WS-M15	600	0.28	0.24	0.04	85.7	18.6
WS-M20	650	0.30	0.25	0.05	83.3	17.4
WS-M25	690	0.34	0.28	0.06	82.4	15.8

S_{BET}: Specific surface area; Brunauer–Emmett–Teller (BET) method.
V_{Total}: Total pore volume; The amount adsorbed $P/P_0 = 0.99$.
V_{Micro}: Micropore volume; Pore volume of micropores was calculated by t-plot.
V_{Meso}: Mesopore volume; V_{Total}-V_{Micro}.
Micropore ratio; $V_{Micro}/ V_{Total} \times 100$.
Yield: $\frac{\text{Walnut shell weight}}{\text{Sample weight}} \times 100$.

Table S3. Structural parameters of walnut shell-based activated carbon as a function of activation time

Sample	002 peak LDCC				002 peak MDCC				10*l* peak		
	2θ (°)	FWHM (2θ)	d002 (Å)	L_c (Å)	2θ (°)	FWHM (2θ)	d002 (Å)	L_c (Å)	2θ (°)	FWHM (2θ)	L_a (Å)
Char	21.26	7.39	4.17	10.82	24.65	5.62	3.61	14.31	43.64	5.36	32.67
WS-M10	21.59	7.02	4.11	11.39	24.65	5.64	3.61	14.27	43.60	4.69	37.30
WS-M15	22.01	6.94	4.03	11.54	24.42	5.51	3.64	14.59	43.64	4.60	38.04
WS-M20	22.02	6.54	4.03	12.25	24.55	5.44	3.62	14.78	43.56	4.05	43.23
WS-M25	22.55	6.37	3.94	12.58	25.07	5.24	3.55	15.35	43.41	3.66	47.76

Table S4. The correlation parameters by adsorption isotherms model

Sample	Langmuir			Freundlich			Langmuir–Freundlich			
	K_L	q_m	R^2	K_F	n	R^2	q_m	K_L	n	R^2
WS-M10	0.941	17.785	0.9942	8.112	4.022	0.9616	29.972	0.325	1.756	0.9999
WS-M15	1.006	18.118	0.9921	8.049	3.899	0.9636	33.067	0.297	1.847	0.9999
WS-M20	0.784	19.687	0.9942	8.347	3.718	0.9641	35.539	0.272	1.720	0.9998
WS-M25	0.517	21.696	0.9942	7.849	3.291	0.9688	44.552	0.186	1.648	0.9997
